# What Is the Difference Between Observed Association and Causal Association, Signals and Evidence? Examples Related to COVID-19

**DOI:** 10.3389/fphar.2020.569189

**Published:** 2021-01-29

**Authors:** Vicki Osborne, Saad A. W. Shakir

**Affiliations:** ^1^ Drug Safety Research Unit, Southampton, United Kingdom; ^2^ School of Pharmacy and Biomedical Sciences, University of Portsmouth, Portsmouth, United Kingdom

**Keywords:** COVID-19, association, causation, signal, evidence

## Introduction

There is a continuous need to identify safe, effective treatments and vaccines which will have a significant impact. However, data can be misinterpreted because of confusion over terminology. We attempt to clarify the difference between observed association and causal association, in addition to the difference between signals and evidence, with examples that have arisen during the COVID-19 pandemic.

### Association and Causation

Determining if there is an association between an exposure and an outcome is one of the fundamental goals in all biomedical research. Specifically in medicine, examining the association between a drug (exposure) and subsequent adverse or beneficial events (outcomes) is one area of interest. However, establishing an association between a drug and an event is not the end of the story but this is where confusion can often arise. The true question of interest is often whether taking the drug causes the event, which can not be established solely because an association is observed. Observed association (the event occurs after taking the drug) is not equivalent to causal association (the event is caused by taking the drug). The reason for this is that there can be other explanations for why a drug appears to be associated with an event; chance, bias and confounding can all play a role. To provide an example in COVID-19, there are many observational studies which have examined the use of experimental treatments and their association with recovery from the disease. However, there could be many possible explanations for the patient’s recovery and recovery is not necessarily because of using those experimental treatments. It is important to ensure that any observed associations are not due to other causes.

In interpreting exposure and outcome data from studies, consideration should be given to the study design being examined. Causal association can often be elucidated from randomised controlled trials (RCTs) because the study design minimizes biases and confounding. The role of chance may still be a factor though. Observational studies can be subject to bias and confounding depending on the design, so should be interpreted with caution. Methods for assessing causal association do exist however, such as the concepts set out by Austin Bradford Hill (Hill, 2015) and subsequent suggested modification (Fedak et al., 2015). Other approaches include causal inference methods which use algorithms and other statistical methods to assess causal associations. Experimental treatments in COVID-19 under investigation include novel therapies such as convalescent plasma; theoretically, the plasma of those who have recovered from COVID-19 may contain sufficient antibodies to treat a patient currently infected with COVID-19 (Chen et al., 2020). Several studies have been completed so far; some of these have not been useful due to small sample sizes and/or lack of randomization or robust study design, so it could not be confirmed that any association between convalescent plasma and recovery was causal (Duan et al., 2020; Shen et al., 2020). Data from ongoing clinical trials which can determine causal association have been inconsistent to date, so a conclusion regarding a causal association is still not possible at this time (Chai et al., 2020). Another example in COVID-19 is hydroxychloroquine, which was originally thought to be a possible treatment because an association with viral clearance was observed in one small study (Gautret et al., 2020). However, there were concerns about the study design (Voss, 2020) and results from a larger RCT revealed no clinical benefit for hydroxychloroquine in COVID-19 (Horby and Landray, 2020). The initial observed association was not found to be causal.

### Signals and Evidence

Signals can arise when examining events that occur after taking drugs (from an observed association). The European Medicines Agency (EMA) defines a safety signal as “Information on a new or known adverse event that is potentially caused by a medicine and that warrants further investigation” (EMA, 2020). Signals are important to identify but are often considered to be hypothesis generating and require further hypothesis testing to provide evidence, which includes evidence of a causal association. Signals are not equivalent to evidence for this reason. They can arise from a single case report or from several spontaneous suspected adverse drug reaction reports. Signal strengthening occurs through assessment of available data related to the signal. In the vast majority of cases a signal alone is not evidence, which must arise from well designed studies e.g., observational or RCTs.

Within the context of COVID-19, safety signals have been identified and investigated further for angiotensin II receptor blockers or angiotensin-converting enzyme inhibitors (ARBs/ACEIs). Due to their mechanism of action, concerns were raised over the potential for predisposition to COVID-19 infection, accelerated coronavirus replication and aggravated symptoms of pneumonia with ARB/ACEI treatment (Guo et al., 2020; Yang and Meng, 2020). Discontinuation of these treatments in patients with hypertension has been suggested as a result. However, results from two retrospective studies in China indicated a lower risk of mortality in COVID-19 patients with hypertension using ARBs/ACEIs compared to non-users. This evidence suggests that discontinuation of ARBs/ACEIs is unlikely to be beneficial (Yang et al., 2020; Zhang et al., 2020). While potential signals should always be investigated further, this should not be considered evidence of a safety concern. Similarly, signals of treatment effectiveness (or lack of effectiveness) should be treated with the same caution. Many experimental treatments are currently under investigation for use in COVID-19 treatment (Thorlund et al., 2020), with case reports on patients who have recovered following treatment (Holshue et al., 2020; Michot et al., 2020). However, this cannot be considered evidence of effectiveness until robust data from ongoing clinical trials become available. Further, there has been much publicized anticipation that vaccines in development for COVID-19 could show “signals” of efficacy prior to final study results (Burger, 2020; Cohen, 2020). Again, it is important to remember that even if such a signal is found, further clinical trial data over a pre-planned data collection period are necessary to provide robust evidence, in addition to further studies in the post-marketing period.

## Conclusion

Despite the need to identify effective and safe treatments as rapidly as possible in the current crisis, it is important to make clear distinctions between observed associations and causal associations. Additionally, consideration should be given to whether exposure and outcome data arise from study designs that provide actual evidence of a causal association or whether they only indicate an observed association (a signal) that requires further investigation. These distinctions are important to ensure understanding and avoid dangerous misinformation.

## Author Contributions

All authors listed have made a substantial, direct, and intellectual contribution to the work and approved it for publication.

## Funding

The Drug Safety Research Unit is an independent charity (No. 327206), which works in association with the University of Portsmouth. It receives unconditional donations from pharmaceutical companies. The companies have no control on the conduct or the publication of the studies conducted by the DSRU.

## Conflict of Interest

The authors declare that the research was conducted in the absence of any commercial or financial relationships that could be construed as a potential conflict of interest.
